# Assessing phytotoxicity and tolerance levels of ZnO nanoparticles on *Raphanus sativus*: implications for widespread adoptions

**DOI:** 10.3762/bjnano.15.11

**Published:** 2024-01-23

**Authors:** Pathirannahalage Sahan Samuditha, Nadeesh Madusanka Adassooriya, Nazeera Salim

**Affiliations:** 1 Department of Botany, Faculty of Applied Sciences, University of Sri Jayewardenepura, Nugegoda, Sri Lanka; 2 Department of Chemical and Process Engineering, Faculty of Engineering, University of Peradeniya, Peradeniya, Sri Lanka

**Keywords:** phytotoxicity, *Raphanus sativus*, ZnO nanoparticles, Zn tolerance, Zn toxicity

## Abstract

The escalating release of zinc oxide nanoparticles (ZnO NPs) into the environment poses a substantial threat, potentially leading to increased concentrations of zinc (Zn) in the soil and subsequent phytotoxic effects. This study aimed to assess the effects of ZnO NPs on *Raphanus sativus* (*R. sativus*) concerning its tolerance levels, toxicity, and accumulation. ZnO NPs were synthesized by the wet chemical method and characterized by powder X-ray diffraction (PXRD), Fourier-transform infrared (FTIR) spectroscopy, ultraviolet–visible (UV–vis) spectroscopy, dynamic light scattering (DLS), and scanning electron microscopy (SEM). The effect of ZnO NPs (70 nm) on *R. sativus* grown in coir was evaluated. The application of 1,000 mg/L of ZnO NPs resulted in a significant increase (*p* < 0.05) in soluble protein content, carbohydrates, chlorophyll a (Chl-a), chlorophyll b (Chl-b), total chlorophylls, carotenoids, and antioxidants by 24.7%, 58.5%, 38.0%, 42.2%, 39.9%, 11.2%, and 7.7%, respectively. Interestingly, this dose had no impact on the indole acetic acid (IAA) content. Conversely, the use of 2,000 mg/L of ZnO NPs in the same medium led to a significant reduction (*p* < 0.05) in soluble protein content by 23.1%, accompanied by a notable increase in IAA by 31.1%, indicating potential toxicity. The use of atomic absorption spectroscopy confirmed the internalization of zinc in seedlings, with a statistically significant increase (*p* < 0.05). In control plants without ZnO NPs, Zn concentration was 0.36 mg/g, while at the highest ZnO NPs tested dose of 10,000 mg/L, it significantly rose to 1.76 mg/g, causing leaf chlorosis and stunted seedling growth. This suggests potential health risks related to Zn toxicity for consumers. Given the adverse effects on *R. sativus* at concentrations above 1000 mg/L, caution is advised in the application and release of ZnO NPs, highlighting the importance of responsible practices to mitigate harm to plant life and consumer health. The study demonstrated the tolerance of *R. sativus* to high Zn levels, classifying it as a Zn-tolerant species.

## Introduction

Despite zinc (Zn) being recognized as an important micronutrient for all living organisms, exceeding the permissible levels of Zn concentration due to anthropogenic sources can be harmful to flora and fauna as well as to humans [1]. Soils contaminated by Zn from different sources could be determined from a Zn concentration higher than 200 μg/g [2], where fertilizers could be cited as one of the anthropogenic sources that significantly contribute to such elevated levels of Zn [1]. High concentrations of Zn are implicated in the shifting of soil microbial communities and inhibition of microbial enzymes, thereby affecting soil fertility [1]. The excess levels of Zn, disrupting soil homeostasis, negatively affects plants and human health by inducing acute toxicity due to the elevated accumulation of Zn [1,3]. Long-term, high-dose Zn supplementation disrupts copper intake, induces brain cell death, contributes to prostate cancer, and also functions as a gliotoxin and a neurotoxin [3–4]. Conversely, the most common micronutrient deficiency of crop plants is Zn deficiency, which affects over 49% of agricultural lands worldwide, thereby negatively affecting crops grown on calcareous and alkaline (pH > 7) soil in dry and semi-arid regions around the world [5–6]. The mean Zn content of soil ranges between 17–125 µg/g of soil while in Zn-deficient soils it is less than 10 µg/g [2]. This suggests the use of organic and inorganic Zn fertilizers to address and alleviate the Zn deficiency and enhance crop yields in Zn-deficient soils [2]. However, when Zn fertilizers are added to soils, Zn gradually changes from the more reactive, readily absorbable, plant-available forms to more stable and less available solid forms by forming complexes with oxides such as aluminum or iron, or by precipitating as Zn carbonate [7]. The recommended Zn level needed for the majority of crops to be healthy ranges from 30 to 200 μg Zn g^−1^ dry weight (DW) of plants [8]. Therefore, the deficiency or excess of Zn can lead to a cascade of metabolic processes that are detrimental to the health of plants as well as humans and other organisms [9]. Hence, there is a need for a better alternative to provide plants with the proper Zn concentration.

Under such circumstances, designing more efficient, novel sources of Zn fertilizers for cultivated crops through the integration of nanotechnology has been the focus of considerable research in the past decade [10]. Nanoparticles have garnered the interest of researchers, leading to their wide application in agriculture due to their enhanced physical, chemical, and biological characteristics compared to those of bulk materials [11]. The enhanced performance of nanoparticles could be attributed to their high specific surface area-to-volume ratio [10]. The availability of a variety of biological, chemical, and physical methods of synthesis, which may be top-down or bottom-up approaches, has facilitated the synthesis of nanoparticles of differing shapes, sizes and properties [11]. When considering zinc oxide nanoparticles (ZnO NPs) in particular, their low toxicity, high biocompatibility, and low cost [12] have enabled them to be applied as a promising strategy to enhance soil fertility and crop productivity, and as a protective agent against phytopathogens [13–14].

On the other hand, even though ZnO NPs are employed in agriculture to maintain sustainability and increase the effectiveness of agricultural activities, the potential toxicity of nanoscale agrochemicals and the unknown risks to the environment and humans have been gaining greater attention [9]. The application of ZnO NPs to the soil as fertilizers and pesticides has given rise to increasing usage of ZnO NPs in consumer products and agriculture [15]. The global yearly output of ZnO NPs is estimated to be between 550–35,000 tons [16]. From this, a significant percentage of approx. 8–28 tons is being released directly or indirectly into the soil environment, where plants are exposed to the direct influence of ZnO NPs [16]. Thus, plants become a prospective conduit for nanoparticle uptake, transport, and bioaccumulation in the food chain when ZnO NPs reach the soil, which positively or negatively affects plant growth and productivity [16]. For instance, ZnO NPs at an optimum concentration of 0.13 g/L promoted seed germination and root growth of groundnut (*Arachis hypogaea* L.) [17]. Similarly, a concentration of 10 mg/L of ZnO NPs elicited a positive response on the root elongation of *Zea mays* (corn). However, at a higher concentration of 1000 mg/L, there was an inhibitory effect on the root elongation of both corn and *Cucumis sativus* (cucumber) seeds, suggesting toxicity associated with elevated ZnO NP concentrations [18]. Hence, this has prompted research into phytotoxic effects of Zn on crops due to the large input of ZnO NPs to the soil from anthropogenic sources [15].

The previously published work on the effect of macronutrients (i.e., hydroxyapatite nanoparticles (HANPs)) on *Raphanus sativus* (radish) with respect to seedling growth and two plant metabolites, serves as the foundational bedrock upon which the current study was built [19]. The insights and discoveries obtained from our earlier research, where a concentration of 10,000 mg/L of raw HANPs had no toxic effects on *R. sativus,* [19] have laid a solid groundwork, providing essential context and understanding to investigate how a micronutrient (i.e., ZnO NPs at high doses) could affect *R. sativus*. Despite the limited commercial utilization of ZnO NPs thus far, there is a concern that once widespread adoption occurs, there may be an excessive release of these nanoparticles. Hence, considering this potential scenario, the main objective of the present study was to investigate the effect of high concentrations of wet-chemically synthesized ZnO NPs in the range of 0–10,000 mg/L on *R. sativus*. Initially, the synthesized ZnO NPs were characterized via several techniques such as powder X-ray diffraction (PXRD), Fourier-transform infrared (FTIR) spectroscopy, solid-UV–vis spectroscopy, dynamic light scattering (DLS), and scanning electron microscopy (SEM). Then the potential phytotoxicity of the synthesized ZnO NPs at higher doses was investigated against *R. sativus* to determine its tolerance. Further, this study also attempted to assess the accumulation of Zn in *R. sativus* seedlings by determining the effect of ZnO NPs on the soluble protein content, indole acetic acid (IAA) content, total carbohydrate content, photosynthetic pigment content, as well as the antioxidant capacity of the leaves of *R. sativus* grown in coir pots. Most of the literature currently reports physiological changes resulting from the application of minute quantities of ZnO NPs. This study demonstrates that *R. sativus* can withstand high doses of Zn levels, thereby being identified as a Zn-tolerant species, ultimately releasing an excess amount of Zn into the environment.

## Results

### ZnO NPs characterization

**Figure 1:**
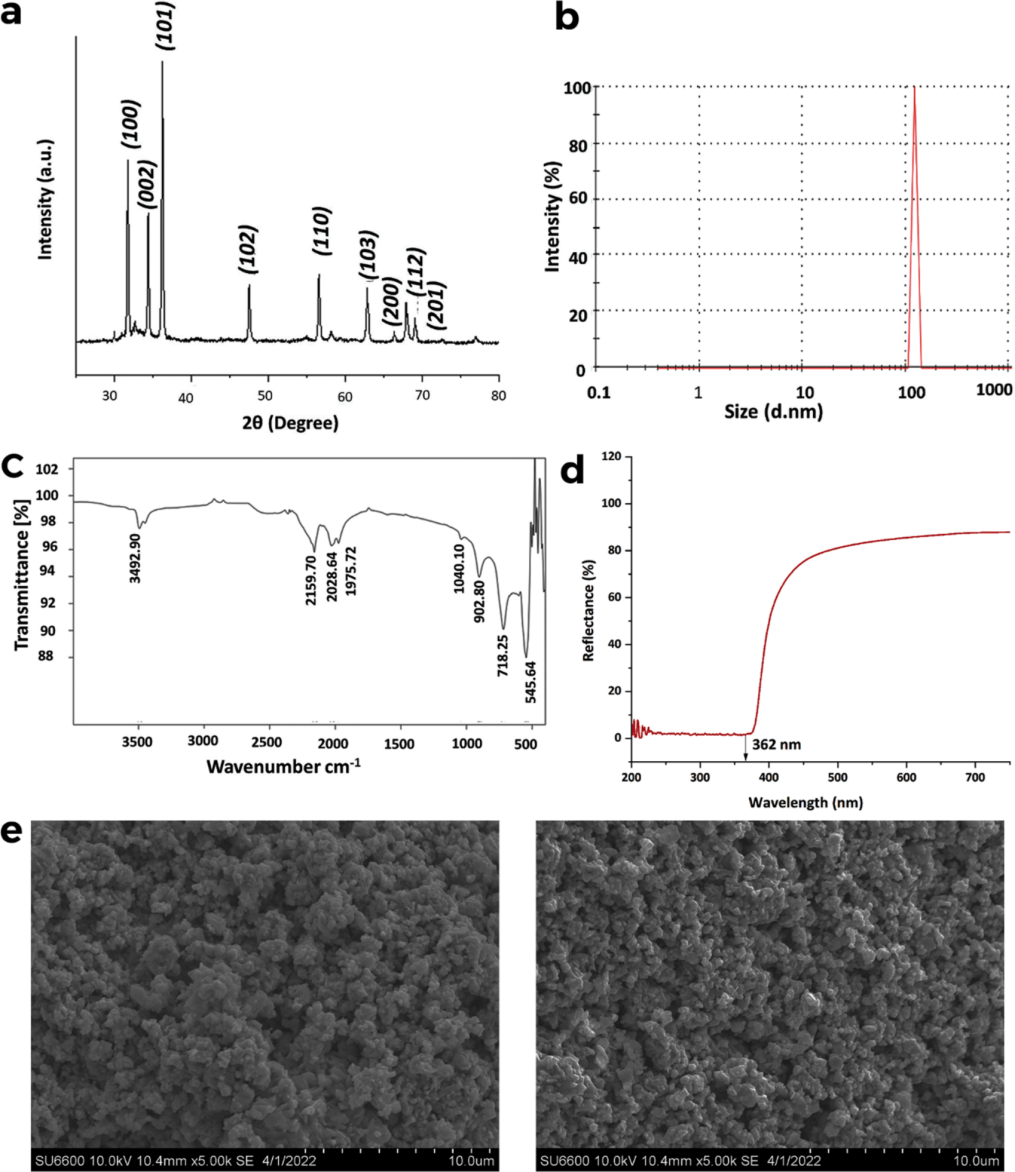
(a) The PXRD spectrum, (b) DLS distribution, and (c) FTIR spectrum. (d) Solid-UV–vis spectrum and (e) SEM images of synthesized ZnO NPs by the wet chemical method.

Wet chemical synthesis yielded 7.012 g of ZnO NPs, disregarding the sample loss during the synthesis. The XRD pattern corresponds to the hexagonal wurtzite structure and is consistent with the Inorganic Crystal Structure Database card number (ICSD card No. 067454) for the pure ZnO phase with space group P63mc (Figure 1a). All the diffraction peaks at angles (2θ) of 31.77°, 34.43°, 36.26°, 47.55°, 56.61°, 62.89°, 66.39°, 67.98°, and 69.10° correspond to the reflection from (100), (002), (101), (102), (110), (103), (200), (112), and (201) crystal planes of ZnO NPs, respectively. The average size reported in the particle size analyzer for ZnO NPs was 122.4 nm, as shown in Figure 1b, with a polydispersity index of 0.332. The FTIR spectrum of ZnO NPs (Figure 1c) shows significant absorption peaks at 545, 718, 902, 2028, and 2159 cm^−1^. The UV–vis spectrum of synthesized ZnO-NPs displays a broad band at 362 nm (Figure 1d). The SEM images (Figure 1e) confirmed that ZnO NPs have spherical morphology with an average diameter of 70 nm.

### Phytotoxicity of ZnO NPs on *R. sativus* grown in an inert solid medium

**Figure 2:**
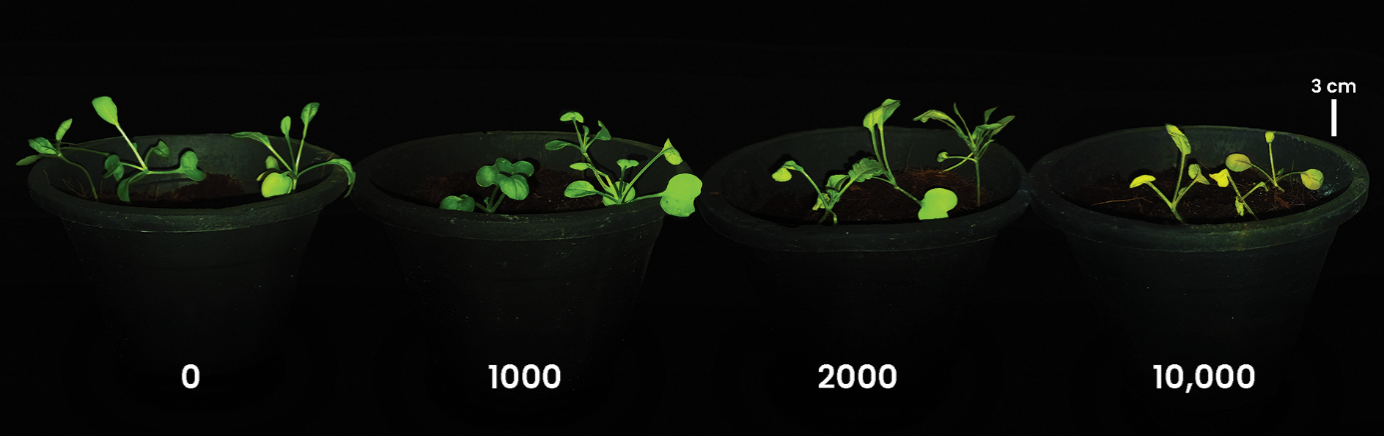
*R. sativus* seedlings treated with 0, 1000, 2000, and 10,000 mg/L of ZnO NPs in combination with Hoagland solution in coir after 30 days of growth. Notably, the seedlings under the 10,000 mg/L treatment displayed wilting and chlorosis and did not survive for 45 days.

*R. sativus* grown on coir fiber medium at 10,000 mg/L of ZnO NPs died showing wilting and yellowing symptoms (Figure 2) and could not survive for 45 days. Therefore, the experiment was repeated using a lower range of ZnO NPs (0–2000 mg/L) in the same medium and the phytotoxicity was evaluated with respect to soluble protein and IAA contents.

#### Effect of ZnO NPs on the soluble protein content

**Figure 3:**
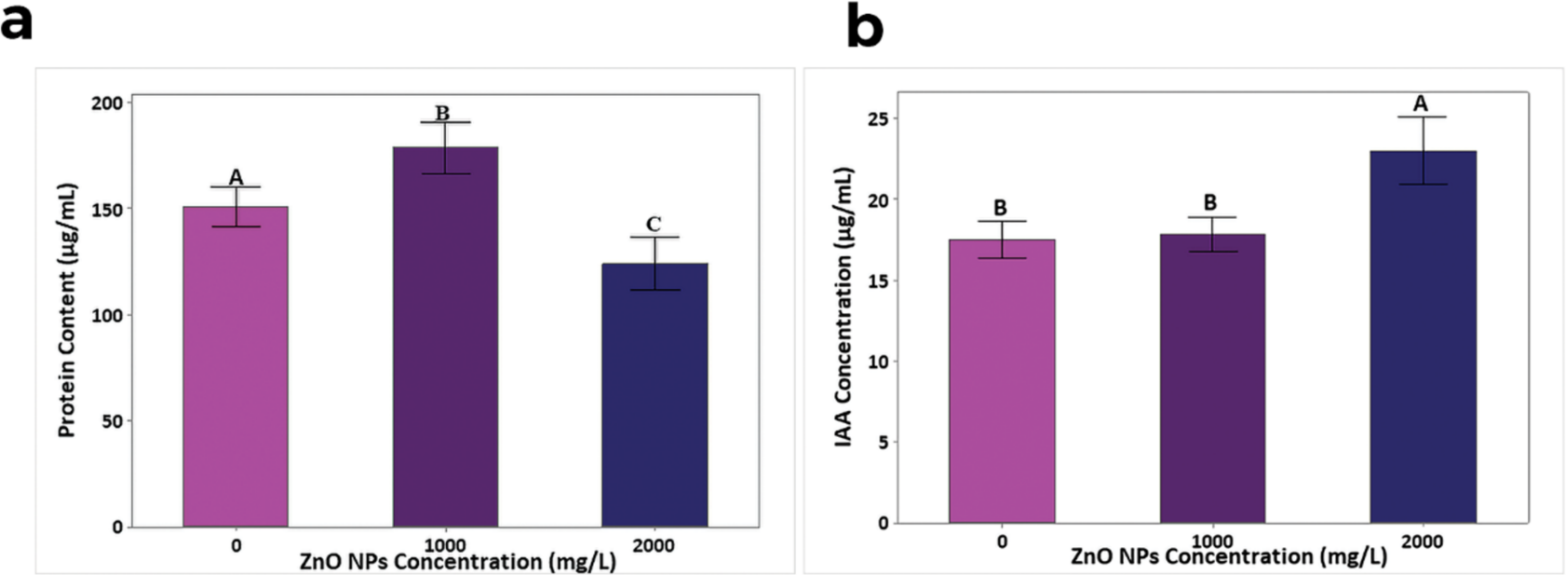
(a) The soluble protein content and (b) IAA content (mean ± SD, *n* = 3) in leaves of 45-day-old *R. sativus* treated with 250 mL of 1000 and 2000 mg/L of ZnO NPs + Hoagland solution compared to that of plants without ZnO NPs in inert coir medium. Treatments with the same letter are not significantly different in their means at *p >* 0.05.

The results revealed that 2000 mg/L of ZnO NPs reduced the soluble protein content by 23.1% while it significantly enhanced the same by 24.7% (*p* < 0.05) at 1000 mg/L of ZnO NPs compared to that of the control (Figure 3a).

#### Effect of ZnO NPs on the IAA content

The ZnO NPs significantly increased the IAA content in the leaves of *R. sativus* (*p* < 0.05) by 31.1% at the dose of 2000 mg/L (Figure 3b). However, no significant (*p* > 0.05) change was observed at 1000 mg/L of ZnO NPs, although a still higher (1.7%) IAA content was produced at 1000 mg/L without exhibiting any toxicity.

### Phytotoxicity testing for carbohydrates, plant pigments, and antioxidant contents

**Figure 4:**
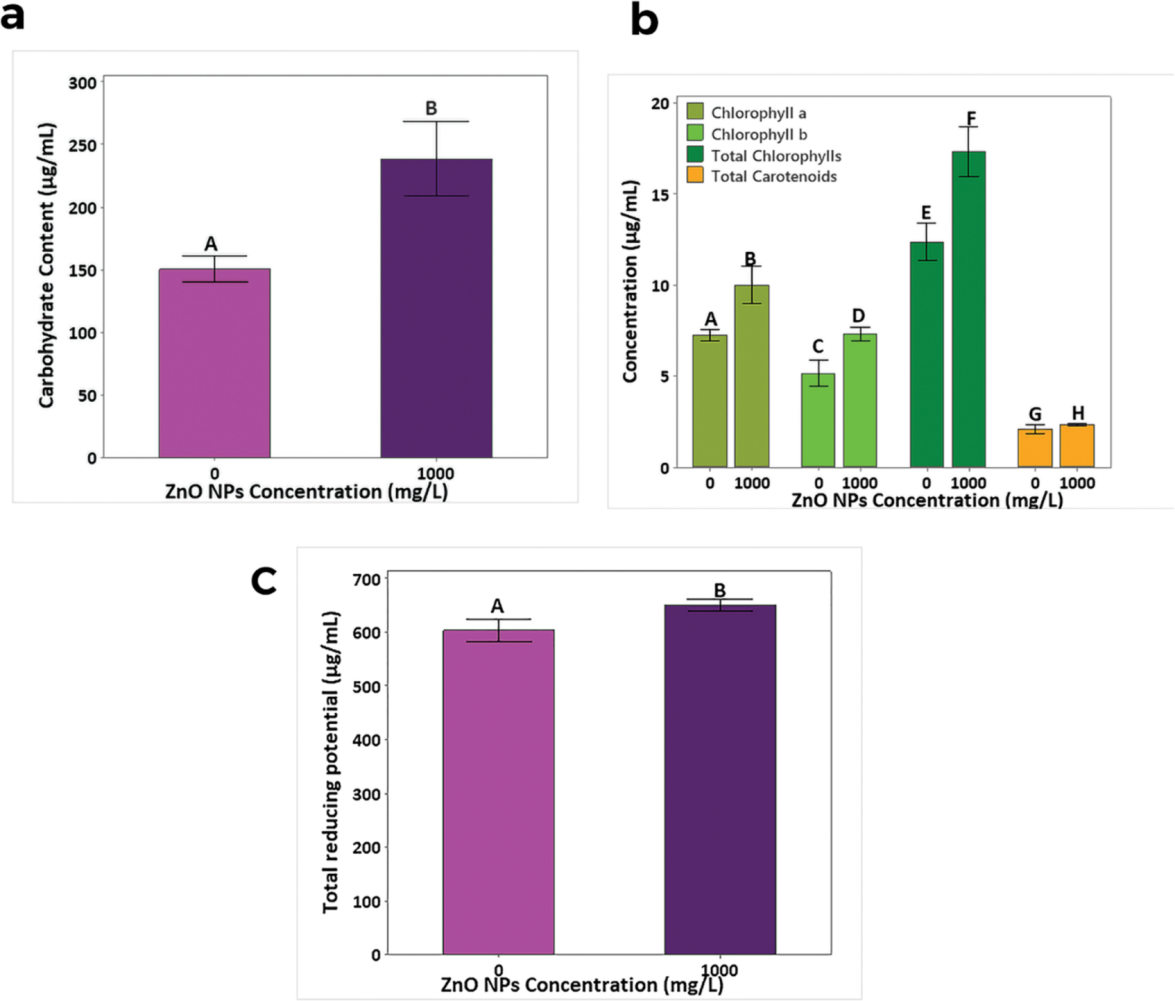
(a) The carbohydrate, (b) plant pigment, and (c) antioxidant contents (mean ± SD, *n* = 3) in the leaves of 45-day-old *R. sativus* grown in coir medium and treated with 1000 mg/L of ZnO NPs + Hoagland solution in inert coir medium. Treatments with the same letter are not significantly different in their means at *p >* 0.05.

The previous experiment indicated that 1000 mg/L of ZnO NPs was nontoxic with respect to soluble protein and IAA contents. Thus, the same concentration (1000 mg/L) was selected to investigate its effects on carbohydrates, plant pigments, and antioxidant contents. The results revealed that 1000 mg/L of ZnO NPs with Hoagland solution significantly increased (*p* < 0.05) the total carbohydrate content (58.5%, Figure 4a), chlorophyll a (Chl-a) (38.0%, Figure 4b), chlorophyll b (Chl-b) (42.2%), total chlorophyll (Chl 39.9%), carotenoid concentrations (11.2%), and antioxidant content (7.7%, Figure 4c) in the leaves of *R. sativus* compared to those of control plants treated with Hoagland solution without ZnO NPs.

### Zn contents in seedlings

**Figure 5:**
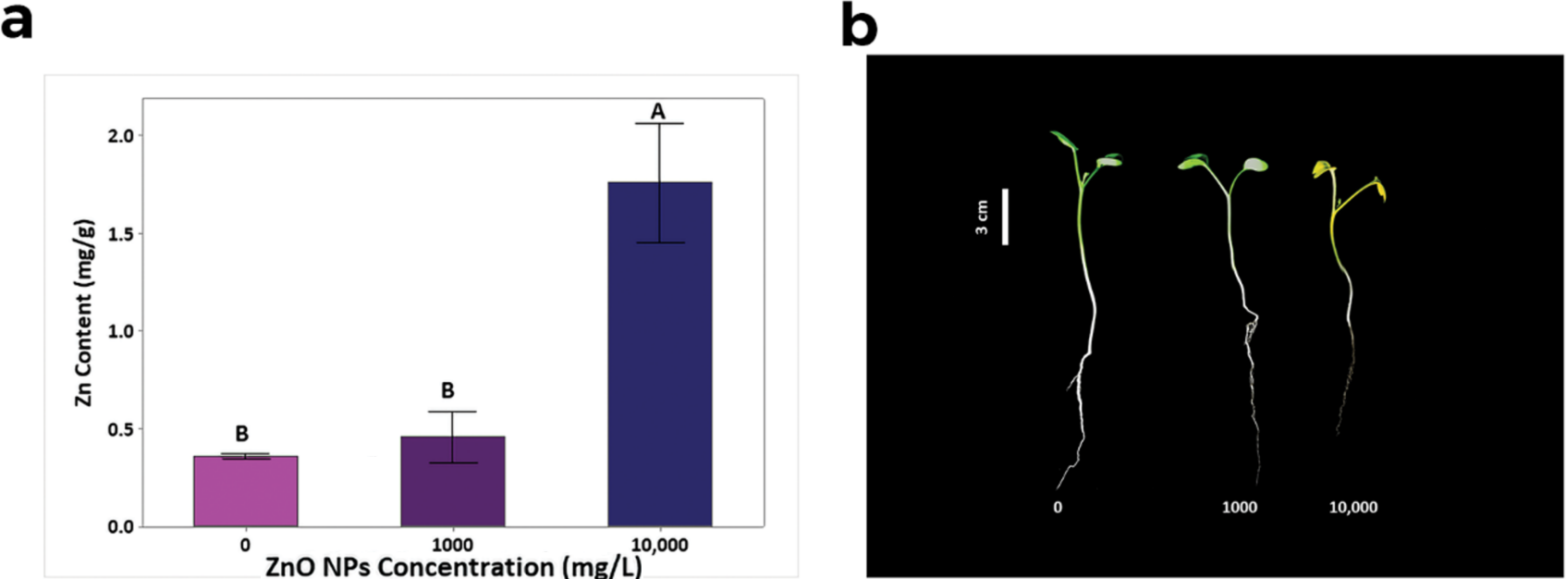
(a) The seedlings of *R. sativus* grown at 0, 1000, and 10,000 mg/L of ZnO NPs. (b) The Zn content per seedling (mean ± SD, *n* = 3) of 18-day-old *R. sativus* treated with 30 mL of 0, 1000, and 10,000 mg/L of ZnO NPs compared to the plants without ZnO NPs. Treatments with the same letter above the bars are not significantly different in their means at *p >* 0.05.

The successful Zn internalization was confirmed by atomic absorption spectroscopy (AAS) analysis (Figure 5a) indicating that *R. sativus* can accumulate Zn at high concentrations (0.36 mg Zn g^−1^ DW of seedlings at 0 mg/L and 1.76 mg Zn g^−1^ DW of seedlings at 10,000 mg/L, *p* < 0.05). Even though the Zn internalization was increased by 27.4% (0.46 mg Zn g^−1^ DW) in the *R. sativus* seedlings treated with 1000 mg/L of ZnO NPs, without showing any adverse effects on morphology (Figure 5b) compared to that of the control, this increment was not significant (*p* > 0.05).

## Discussion

### ZnO NPs characterization

The PXRD technique provides important insights into the chemical composition, physical characteristics of the material and crystallographic structure, and crystalline particle size based on the scattered X-ray beam intensity [20–21]. The PXRD pattern (Figure 1a) of synthesized ZnO NPs was in accordance with the literature in terms of peak positions and relative intensity [22–23]. The sharp diffraction peaks and primary strong angles indicated the good crystallinity of the prepared crystals [24–25]. Furthermore, the obtained patterns confirmed that no other contaminants were present in the PXRD pattern, specifying that the principal component at the inorganic phase of the sample was ZnO. Hence, the findings unequivocally substantiated the synthesis of ZnO NPs. The FTIR analysis was conducted to validate the presence of specific functional groups on the surface of the synthetic materials [26]. The bonding of Zn–O is in the range of 400–1090 cm^−1^ [27–29]. Therefore, the distinctive bands in the FTIR spectrum at 545–1040 cm^−1^ could be attributed to the stretching vibration of the metal oxide, which belongs to the ZnO metal group [27–30]. The peak at 3492 cm^−1^ is attributed to the characteristic vibrational mode of the O–H bond which comes from water adsorbed by the ZnO NPs from the humid atmosphere [31]. Typically, bulk ZnO particles show a characteristic absorption edge of 400 nm in UV–vis spectra. Hence, ZnO NPs show blue shifting and have an absorption peak below 400 nm due to the nanometric size effect of the synthesized ZnO and characteristic hexagonal ZnO NPs [32]. A broad band at 362 nm in the UV–vis spectrum was reported, indicating the formation of ZnO NPs, and it could be due to an electron transfer from the valence to the conduction band in the main band gap of ZnO, Zn 3d→O 2p [20]. The larger average diameter (122.4 nm) than that of the SEM images (70 nm) is due to the fact that particles in solutions are generally larger than those directly seen via microscopy techniques [33]. The increased average diameter and polydispersity index could be linked to the agglomeration of nanoparticles caused by the rapid addition rate of NaOH during the synthesis process, as evidenced by the presence of agglomerated particles visible in the SEM images [33]. Further, the light scattering technique results are biased toward the larger particles in the sample [34].

### Phytotoxicity experiments on *R. sativus* grown in an inert solid medium

The high bioavailability of Zn caused by the acidic pH (5.8–6.5) of coir might have caused the death of *R. sativus* grown with the application of 10,000 mg/L of ZnO NPs. This is confirmed by leaf chlorosis after 18 days of growth and significant internalization of Zn at 10,000 mg/L by treated plants in comparison with the control group (Figure 5). Soluble protein content has been widely used as a toxicity parameter to evaluate phytotoxicity in different species. As an example, an increase in soluble protein content in olive tree (cv. Moraiolo) shoots in vitro was reported upon treatment with ZnO NPs at concentrations of 6 and 18 mg/L [35].

Zinc is essential for the synthesis of tryptophan, an amino acid required for the biosynthesis of IAA. Zinc has an indirect influence on auxins by activating tryptophan synthase, an enzyme responsible for the synthesis of tryptophan in the biosynthesis of IAA [36–37].

According to the present results, ZnO NPs have no toxic effect on IAA content at 1000 mg/L; however, it increased the IAA content at 2000 mg/L. Zinc oxide NPs are a potential candidate to enhance the nutritional content of *R. sativus*. The positive correlation between chlorophyll content and photosynthetic rate allows the use of changes in total chlorophyll content as an indicator of plant health [38]. The increase in chlorophyll content observed in this study may be attributed to the increase in nutrients and water uptake in the presence of Zn [39], or else by accelerating the activity of carbonic anhydrase [2]. Several published information is available on the effect of Zn on photosynthetic pigments of other crops. Nanopriming *Triticum aestivum* seeds with 10 mg/L of ZnO resulted in a significant enhancement of photosynthetic pigments, including a 48% increase in Chl-a, a 50% increase in Chl-b, a 49% increase in total chlorophyll, and a 34% increase in the carotenoid content compared to those of the control [40]. Further, the application of biosynthesized ZnO NPs has shown the ability to suppress *Fusarium* wilt in *Solanum melongena* L. while enhancing its carbohydrates and chlorophyll contents [13].

In contrast, a significant reduction of chlorophyll fluorescence of *Hordeum sativum* (barley) treated with 2000 mg/L of ZnO NPs compared to 300 mg/L of ZnO NPs has also been reported [41]. These pieces of evidence confirmed that the effect of ZnO NPs on photosynthetic pigments is highly crop-specific and dose-specific.

Many researchers have reported increased antioxidant content when treated with ZnO NPs. For example, increased activity levels of catalase, glutathione reductase, superoxide dismutase, and glutathione S-transferase in *Hordeum vulgare* by 3-fold in roots exposed to 2000 mg/L of ZnO NPs have been reported [42]. Also, Zn is an essential element of carbohydrate metabolism in plants [43]. The highest proportions of carbohydrates (40.3%), fiber (12.0%), and energy (161.2% in kcal/g carbohydrates) were observed in common beans upon the application of ZnO NPs at a concentration of 10 ppm [44].

Therefore, it is apparent that Zn is a positive stimulator of carbohydrates, chlorophyll, carotenoid, and antioxidants synthesis in *R. sativus* at 1000 mg/L of ZnO NP without causing any toxicity.

### Zn content in seedlings

The normal Zn level required for healthy plant growth is 0.015–0.02 mg Zn g^−1^ DW [45]. Hence, the Zn internalization by *R. sativus* at 10,000 mg/L was way beyond (340%) the healthy level (<0.4 mg Zn g^−1^ DW) [46]. Remarkably, *R. sativus* demonstrated the ability to grow at 1000 mg/L of ZnO NPs in coir without displaying chlorosis, necrosis, or strong growth inhibition, indicating its tolerance to elevated Zn levels [47]. Its resilience may have implications not only for the plant but also for the surrounding soil environment, microflora, and potentially human health.

Metal-based engineered nanomaterials may dissolve and then undergo biotransformation or be internalized as intact particles in a biological context [48]. As an example, the biotransformation of ZnO NPs into Zn nitrate, Zn phosphate, and Zn citrate in desert plant species has been reported [49]. Also, the bioaccumulation of intact ZnO NPs (>30 nm to <50 nm) in the intercellular space, vacuole, and cytoplasm of *Triticum aestivum* roots when exposed to 15,000 ppm of ZnO NPs has been reported [50]. However, the average particle size in the present study (70 nm) is beyond the size-exclusion limits (5–20 nm) of the cell wall [51]. Hence, there was probably less chance of intake of intact ZnO NPs than that of Zn^2+^ by *R. sativus*.

Further, upon application or release into the environment, ZnO NPs undergo rapid dissolution and conversion while some NPs attach to the crop root surface [52]. The ability of ZnO NPs to adhere to the root surface is higher than that of bulk ZnO [53]. As an example, the influence of ZnO NPs (15–40 nm) on Zn accumulation in *H. vulgare* indicates a significant increase in the roots, without a corresponding effect in the shoot, suggesting substantial adhesion of ZnO NPs to the root surface [42]. Further, previous studies have also highlighted the significant adherence of ZnO NPs to the root surface of *Lolium perenne* and *Zea mays* [53–54].

## Conclusion

In this study, ZnO NPs were successfully synthesized via a wet chemical method from the ZnCl_2_ precursor and characterized using PXRD, FTIR, UV–vis, and DLS, resulting in crystalline, spherical particles with an average diameter of 70 nm. Application of these ZnO NPs to radish plants in coir with a dose of 1000 mg/L showed no toxicity in terms of soluble protein content, carbohydrates, plant pigments, and antioxidants while significantly enhancing them compared to the control without the ZnO NP treatment. Interestingly, there was no significant change in IAA content at a dose of 1000 mg/L of ZnO NPs. However, the most substantial enhancement of selected metabolites such as soluble protein content, carbohydrates, plant pigments and antioxidants was observed at 1000 mg/L. Conversely, at a concentration of 2000 mg/L, ZnO NPs significantly reduced soluble proteins and increased IAA levels, indicating toxicity and physiological stress. Plants treated with a dose of 10,000 mg/L exhibited wilting and yellowing by day 18 and did not survive until day 45. A noteworthy internalization of Zn by *R. sativus* at 10,000 mg/L exceeded the healthy level of Zn by 340%. Despite this, *R. sativus* demonstrated survival at 1000 mg/L without signs of chlorosis, necrosis, or growth inhibition, suggesting its Zn-tolerant nature. This study emphasized the need for proactive measures to address potential risks associated with increased quantities of ZnO NPs in the environment.

## Experimental

### Synthesis of ZnO NPs

Synthesis of ZnO NPs was done using methanolic solutions of 0.8 M NaOH (250 mL) and 0.4 M ZnCl_2_ (250 mL) [22–23,55]. The ZnCl_2_ solution was strenuously stirred at 4000 rpm in a magnetic stirrer and NaOH was added dropwise, the mixture was kept under constant stirring for 2 more hours and then sealed and kept overnight. The Zn(OH)_2_ obtained from the reaction was collected by centrifugation, washed with distilled water, and calcined at 400 °C for 2 h to obtain ZnO NPs. The concentration ratio between zinc chloride and sodium hydroxide was assessed utilizing the chemical equation formula provided below:




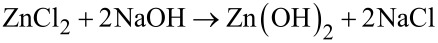




Upon drying, the transformation of Zn(OH)_2_ into ZnO was elucidated by the following equation.




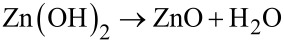




### Characterization of ZnO NPs

Then ZnO NPs were characterized using PXRD (Rigaku Ultima IV X-ray Diffractometer) with Cu Kα radiation (1.54059292 Å) over a 2θ range of 20–80° with a scan speed of 2° min^−1^ at 30 mA and 40 kV [25]. The chemical nature and molecular bonding of the synthesized sample were studied using FTIR (Bruker Vertex80 FT-IR Spectrometer) at a range of 400–4000 cm^−1^ using attenuated total reflection mode. The characteristic absorption band of the ZnO NPs sample was measured by LAMBDA 365 UV Spectrometer at a wavelength range of 200–800 nm. The particle size distribution of ZnO NPs was evaluated by a Malvern Zetasizer at 25 °C with a count rate of 171.1 kcps, a duration of 50 s. Water was used as a dispersant with a NP concentration of 25 ppm following sonication for 30 min at 45 kHz using a GT SONIC-L3 sonicator. Particle size and shape of ZnO NPs were analyzed using Hitachi SU6600 FE (Field Emission)-SEM at the Sri Lankan Institute of Nanotechnology (SLINTEC).

### Phytotoxicity experiments on *R. sativus* grown in an inert solid medium

*R. sativus* was grown in an inert coir medium with three seeds sown in each pot, and weekly treatments were performed as follows [19]: 250 mL of modified Hoagland solution without and with ZnO NPs (0, 1000, 2000, 10,000 mg/L) was added per pot. The treatments were introduced when plants were 14 days old and stopped when the plants were 45 days. The solutions were sonicated for 30 min at 45/65 kHz using a GT SONIC-L3 sonicator before application [19] to disperse NPs agglomerates by generating evacuated cavities or microvoids in the liquid. This exerted a shear force on NP agglomerates, effectively overcoming the van der Waals force that holds them together [56].

#### The effect of ZnO NPs on soluble protein and IAA contents

Plants grown in coir medium treated with 10,000 mg/L did not survive. Therefore, protein and IAA contents were analyzed for those plants treated with 1000 and 2000 mg/L of ZnO NPs. Fresh leaf samples were separately obtained from all three replicates for each analysis; treated with ZnO NP + Hoagland or Hoagland without ZnO NPs (controls).

#### Determination of soluble protein content

Leaf samples (200 mg) were separately homogenized using a mortar and pestle with 10 mL of phosphate buffer (50 mM at pH 7.4). The mixture was centrifuged at 9000 rpm for 15 min at 4 °C, the supernatant was collected, and the soluble protein content was determined according to the Bradford’s method [57].

#### Determination of IAA content

Leaf samples (300 mg of young leaves) were obtained, freeze-dried at −80 °C, and crushed using a chilled mortar and pestle. Then, they were mixed with 5 mL of 80% (v/v) methanol containing 100 mg/L of ascorbic acid as an antioxidant [58]. The mixture was stirred for 10 min and incubated for 48 h in the dark. It was then centrifuged at 3500 rpm at 4 °C and 1 mL of the supernatant was mixed with 2 mL of Salkowski reagent and incubated in the dark at room temperature for 30 min. Then, the absorbance was measured at 530 nm [19,59].

### The effect of ZnO NPs on carbohydrates, plant pigments, and antioxidant levels

*R. sativus* plants treated with 1000 mg/L of ZnO NPs were selected for further biochemical testing.

#### Determination of total carbohydrate content in leaves

The leaf samples (50 mg) were separately homogenized with 5 mL of 80% ethanol and extracted by boiling in a water bath at 95 °C for 10 min [60]. The ethanol extracts of the samples were centrifuged at 2500 rpm for 5 min and the supernatant was analyzed for carbohydrate content by the phenol-sulfuric method [61].

#### Determination of pigment content in leaves

Fresh leaf samples (50 mg) were obtained, and chlorophyll a (Chl-a), chlorophyll b (Chl-b), total chlorophyll, and carotenoid contents were determined [62–63].

#### Determination of antioxidants in leaves

Leaf samples (50 mg) were homogenized using a mortar and pestle after adding 5 mL of distilled water and centrifuged at 3000 rpm for 10 min. An aliquot of 400 µL of supernatants from each sample was taken and the antioxidants in leaves were analyzed by the ferrous reducing antioxidant capacity assay [64].

#### Determination of Zn content in *R. sativus* seedlings

The Zn contents from 18-day-old *R. sativus* seedlings treated with 10,000, 1,000, and 0 mg/L of ZnO NPs were measured with Thermo Scientific iCE 3500 AAS using air-dried seedlings (until obtaining a constant mass). Plants were digested with acid using 7.5 mL of 65% HNO_3_ for 3 h at 90 °C [65]. Finally, 2.5 mL of distilled water was added and filtered with nylon syringe filters (0.2 μm).

#### Data analysis

One-way ANOVA followed by Tukey’s HSD test was employed to examine the statistical differences concerning soluble protein content, IAA, and Zn internalization. A two-sample t-test was employed to examine the statistical differences with respect to carbohydrates, plant pigments, and antioxidant activity. The results were expressed as mean ± SD (standard deviation). Values of *p* ≤ 0.05 were considered to be significantly different. All the statistical analyzes were conducted using Minitab version 21.1.1 (64-bit).
